# Collision of germline *POLE* and *PMS2* variants in a young patient treated with immune checkpoint inhibitors

**DOI:** 10.1038/s41698-022-00258-8

**Published:** 2022-03-08

**Authors:** Enrico Berrino, Roberto Filippi, Clara Visintin, Serena Peirone, Elisabetta Fenocchio, Giovanni Farinea, Franco Veglio, Massimo Aglietta, Anna Sapino, Matteo Cereda, Rosella Visintin, Barbara Pasini, Caterina Marchiò

**Affiliations:** 1 Candiolo Cancer Institute, FPO IRCCS, Candiolo, Italy; 2 Department of Medical Sciences, University of Turin, Turin, Italy; 3 Department of Oncology, University of Turin, Turin, Italy; 4 Medical Oncology Unit, University Hospital AOU Città della Salute e della Scienza, Turin, Italy; 5 Department of Experimental Oncology, European Institute of Oncology, IEO IRCCS, Milano, Italy; 6 Cancer Genomics and Bioinformatics Unit, IIGM-Italian Institute for Genomic Medicine, c/o IRCCS Candiolo, 10060 Turin, Italy; 7 Internal Medicine Unit, University Hospital AOU Città della Salute e della Scienza, Turin, Italy; 8 Medical Genetics Unit, University Hospital AOU Città della Salute e della Scienza, Turin, Italy

**Keywords:** Molecular medicine, Cancer genetics

## Abstract

The onset of multiple and metachronous tumors in young patients induces to suspect the presence of genetic variants in genes associated with tumorigenesis. We describe here the unusual case of a 16-year-old patient who developed a synchronous bifocal colorectal adenocarcinoma with distant metastases. We provide high throughput molecular characterization with whole-exome sequencing (WES) and DNA targeted sequencing of different tumoral lesions and normal tissue samples that led to unveil a germline *POLE* mutation (p.Ser297Cys) coexisting with the *PMS2* c.2174 + 1 G > A splicing mutation. This clinical scenario defines a “POLE-LYNCH” collision syndrome, which explains the ultra-mutator phenotype observed in the tumor lesions, and the presence of MMR deficiency-associated unusual signatures. The patient was successfully treated with immune checkpoint inhibitors but subsequently developed a high-grade urothelial carcinoma cured by surgery. We complement this analysis with a transcriptomic characterization of tumoral lesions with a panel targeting 770 genes related to the tumor microenvironment and immune evasion thus getting insight on cancer progression and response to immunotherapy.

## Introduction

The onset of multiple neoplasms in young patients is highly suggestive of predisposition variants in genes associated with tumorigenesis. The prevalence of these variants, inherited from parents or de novo acquired, reaches ~40% in pediatric cancers^1^. Many of the altered genes associated with multiple tumor predisposition syndromes are ontologically defined as “caretakers”, i.e., capable of repairing DNA damages and errors accumulated during genome replication^2^. Examples are represented by *BRCA1/2* variants in hereditary breast and ovarian carcinomas, *MUTYH* and *NTHL1* variants in multiple colorectal polyps and cancer, and mutations in the mismatch repair (MMR) genes that predispose to Lynch syndrome, with high risk for colorectal cancer (CRC) and extra-colonic neoplasms (endometrium, ovary, ureter, stomach, and pancreas)^3^. Loss-of-function of these genes (mainly *MLH1*, *MSH2*, *MSH6*, and *PMS2*) reduce the post-replicative capabilities of repairing sequence errors inserted by the most active DNA polymerases, polymerase epsilon catalytic subunit A (encoded by *POLE*), and polymerase delta catalytic subunit (encoded by *POLD1*) whose exonuclease domain proofreads and repairs mis-embedded bases^4^, leading to microsatellite instability (MSI). Mutations in this domain cause an inherited multiple polyposis and cancer predisposition^5^ but they can be also somatically acquired. MMR deficiency and *POLE/POLD1* mutations are correlated with hypermutated/MSI and with ultra-mutator phenotypes, respectively^6^, and have gained interest as positive predictors of immunotherapy response^7–10^.

## Results

### Clinical history

**Fig. 1:**
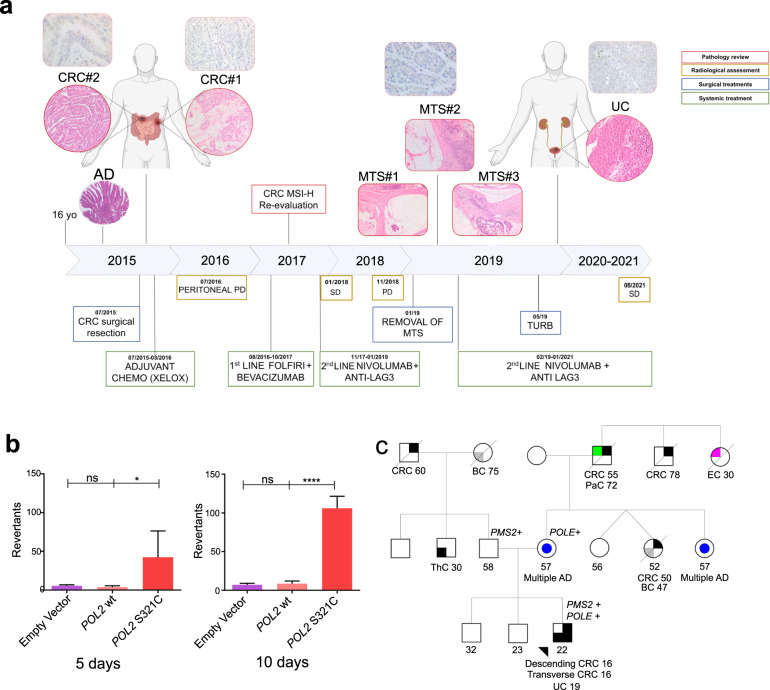
Patient clinical history and validation of the *POLE* variant. **a** Below the time bar surgical events (blue) and treatment regimens (green) are reported. Above the bar, Hematoxylin and Eosin (H&E) and PMS2 IHC images of the histological samples and diagnostic molecular status (red). Circular images: primary carcinomas; rectangular images: metastases. Yellow boxes include RECIST criteria details. **b** *Saccharomyces cerevisiae pol2Δ* haploids auxotrophic for tryptophan (trp1-1) and carrying an empty vector, a centromeric plasmid with the wild-type or *pol2-S312C* allelic variant were plated on media lacking tryptophan to probe for their ability to revert the amber *trp1-1* mutation, thus permitting the growth of revertants in tryptophan-deficient media. Two independent clones per 2 × 10^7^ cell divisions were tested in triplicate for each strain. Errors bars show the mean standard deviation of two biological replicates (3x technical replicates of each genotype). *P* value was calculated with one-way ANOVA and Tukey’s multiple comparisons test (significant *P < 0.05; ****P < 0.0001). **c** Family Pedigree, with the *PMS2* and the *POLE* variants reported. AD adenoma, BC breast cancer, CRC colorectal cancer, EC endometrial cancer, PaC pancreatic carcinoma, MTS metastasis, PD progression disease, SD stable disease, ThC thyroid cancer, TURB trans-urethral resection of the bladder, UC urothelial carcinoma.

A 16-year-old male patient was diagnosed with a synchronous bifocal CRC [transverse (7 cm) and descending colon (4 cm)]. Surgical resection led to a final diagnosis of adenocarcinoma with prominent mucinous features in one of the two lesions, pT4a(m) pN1b (Fig. 1a). An adenomatous polyp of the sigma had been surgically resected some months before this diagnosis.

Following an initial negative MSI result (from BAT25, BAT26, NR21, NR22, and NR24 loci evaluation), the patient received adjuvant chemotherapy (XELOX regimen). He was referred to genetic counseling that revealed a mother with multiple colorectal polyps and other relatives within the maternal family with colorectal polyps, CRC, breast, endometrial and pancreatic cancer. The paternal branch reported relatives with breast cancer, a CRC, and a thyroid cancer diagnosis. The proband was evaluated for mutations in the major CRC predisposing genes (*APC*, *MUTYH*, *MLH1*, *MSH2*, and *MSH6*). A single rare variant in the *APC* gene (c.6694 C > G, p.His2232Asp) was identified but transmitted from the healthy father and classified as a variant of unknown functional significance (VUS, class 3).

One year after surgery the patient developed peritoneal progression and the combination therapy FOLFIRI plus bevacizumab was administered. Primary tumors were reanalyzed for MSI by using both mononucleotide (BAT25, BAT26, NR21, NR22, MONO27, and BAT40) and dinucleotide (D18S58, D2S123, D17S250, and D5S346) loci, revealing a peculiar MSI pattern with three mononucleotide and two dinucleotide loci characterized by a small allelic shift with respect to the constitutional DNA, thus allowing treatment with a combination of two immune checkpoint inhibitors (ICIs), nivolumab and anti-lymphocyte-activation gene 3 (anti-LAG3) antibody (Fig. 1a). Despite a first overall disease control with maintained stable disease (SD) as best response according to RECIST criteria v.1.1, the patient developed an isolate progression (PD) on a pelvic peritoneal node after 12 months of treatment and underwent surgical resection of the pelvic progressive metastasis together with three other peritoneal nodes, all histologically confirmed as adenocarcinoma of mucinous type. The patient resumed treatment with nivolumab + anti-LAG3 antibody.

One and a half year following the inclusion in the treatment protocol, the patient was diagnosed with a noninvasive high-grade urothelial carcinoma of the bladder, which was surgically removed (pTa) (Fig. 1a).

At present, the patient is still on ICI treatment and free of disease.

### Identification of germline variants

Whole-exome sequencing (WES) from blood resulted in a mean read-depth of 300X. To identify pathogenic variants, InterVar was interrogated and data interpreted with a two-step approach. First, we scanned for variants with known clinical significance and WES annotation identified 56 ClinVar pathogenic or likely pathogenic alterations, and five related to cancer. Of these, the *PMS2* c.2174 + 1 G > A was a rare, Lynch-related splicing variant of exon 12. The pathogenicity of this mutation has been previously reported^11^. The targeted OCAv3 panel was applied on the germline DNA and this heterozygous variant was confirmed. IHC analysis demonstrated that all of the lesions lacked PMS2 expression (Fig. 1a).

Second, we screened for the rarest variants in the hereditary CRC-associated genes. This approach revealed the already known missense VUS within the *APC* gene (c.6694 C > G, p.His2232Asp) and an exonuclease domain variant of *POLE* p.Ser297Cys (c.890 C > G). The TSO500 panel confirmed the heterozygosity of the *POLE* p.Ser297Cys alteration.

To elucidate the behavior of the *POLE* variant, we first interrogated different predictor tools. Of note, the substitution of the highly conserved, polar Serine with the hydrophobic Cysteine was relevant for both the BLOSUM62 matrix and the Grantham distance. In addition, 10 out of 13 prediction tools revealed a non-tolerated behavior, also confirmed by the stand-alone A-GVGD tool (class 65). Finally, the Phyre2 3D analysis revealed a slight influence over the protein stability, confirming the “structural tolerability” of the variant as expected for *POLE* germline mutations associated with cancer and polyp predisposition^5^.

Despite the absence of the variant in public germline datasets, somatic mutations at the same amino acid residue were described as pathogenic on the basis of the ultra-mutator phenotype of the corresponding tumors^12,13^. To validate the pathogenicity of this variant, we conducted a functional assay in the budding yeast *Saccharomyces cerevisiae*. For this purpose, we used the *trp1-1* allele of the *TRP1* gene. The *trp1-1* allele carries a GAG to TAG amber nonsense change at codon 83^14^. We transformed a heterozygous *POL2/pol2Δ* diploid strain, auxotrophic for tryptophan (*trp1-1/trp1-1*), with an empty vector or with a centromeric plasmid carrying either the wild-type *POL2* gene or the *pol2*-Ser312Cys variant. *POL2* is the yeast ortholog of human *POLE* and *pol2*-Ser312Cys corresponds to the human *POLE*-Ser297Cys. Next, exploiting the ability of yeast to switch between mitosis and meiosis and to live both in haploid and diploid statuses, we assessed the variant ability to rescue *pol2Δ* lethality and its mutagenic potential. The *pol2* variant sustained viability as the wild-type (Supplementary Fig. 1) but it was highly mutagenic—as assessed by the increased number of revertants of the *amber trp1-1* mutation capable of growing in tryptophan-deficient media—when present in homozygosis (Fig. 1b). Instead, the phenotype associated with the mutation was not penetrant in an otherwise wild-type background if the wild-type *POL2* gene was also present (data not shown). These data support the hypothesis that the p.Ser297Cys *POLE* variant holds a pathogenic potential.

Finally, parent germline DNA was sequenced with both TSO500 and OCAv3 panels to elucidate the inheritance from the paternal or maternal branch of the two variants. We confirmed the maternal transmission of the *POLE* mutation (by both panels), whereas the *PMS2* splicing variant was transmitted from the father and identified only by the OCAv3 panel since the TSO500 does not cover the exon 12 (Fig. 1c).

### WES and somatic mutation analysis

The mutational somatic landscape of the primary CRCs and metastases was assessed both with WES and the TSO500 targeted panel. The former sequencing approach was used to predict the mutational signatures whereas the latter performed high-deep sequencing on cancer-related genes for variant calling.

**Fig. 2:**
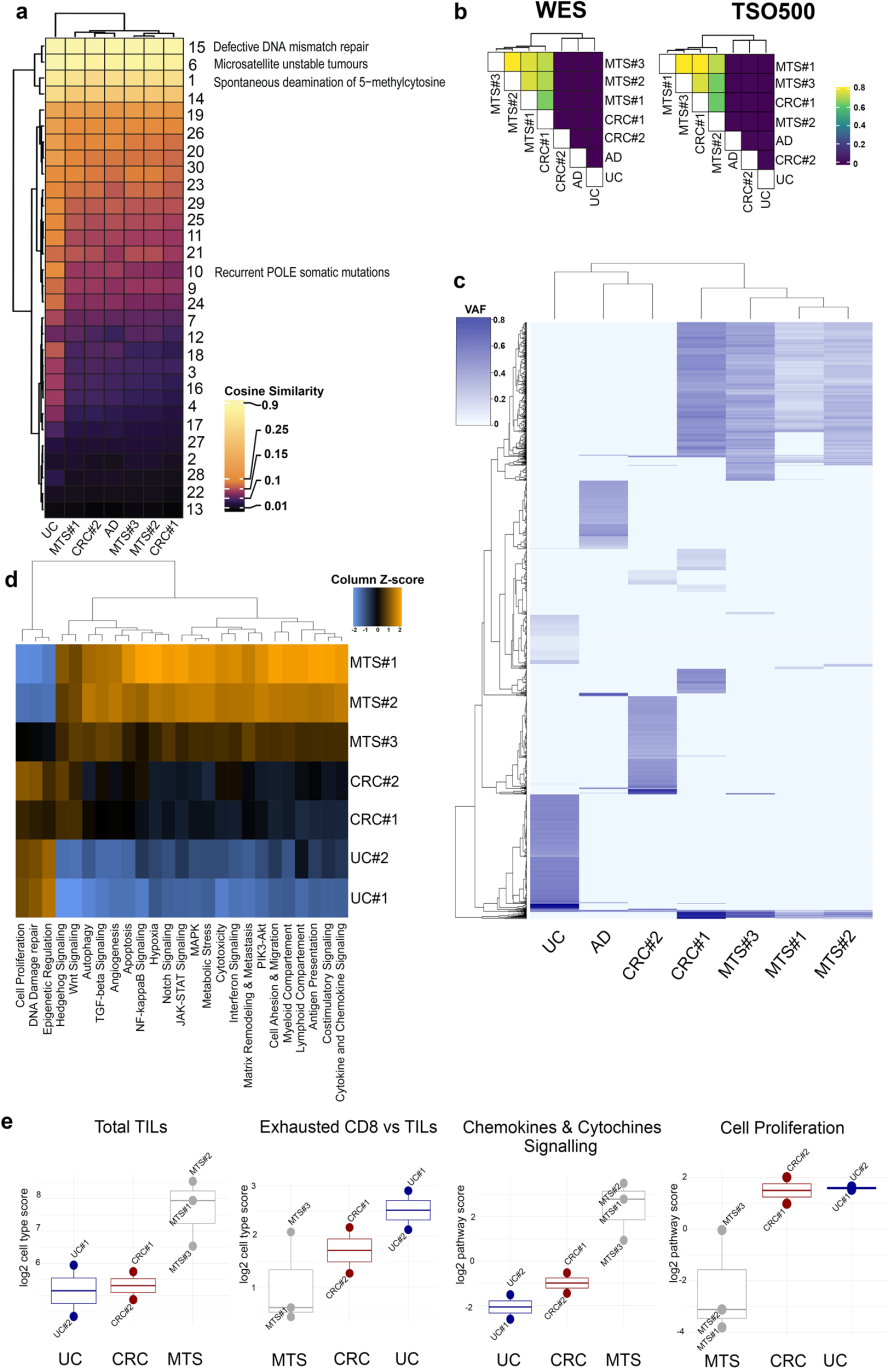
Genetic and transcriptomic analysis of primary and metastatic lesions. **a** Mutational signature results from WES. The heatmap shows the prevailing Signatures 15, 6, 1, 14 in all the lesions. **b** Similarity matrix for mutated genes identified by WES and TSO500 panel for all the sequenced lesions. The Jaccard coefficient was the proportion of shared altered genes over the total number of altered genes. **c** Unsupervised VAF heatmap for somatically identified variants. **d** *Z*-scored “pathway scores” heatmap derived from gene expression analysis. **e** Box plots (min to max and 25, 50, 75 percentiles) of the cell types and pathway scores. Circles represent different samples. CRC#1: mucinous colorectal adenocarcinoma; CRC#2: non-mucinous colorectal adenocarcinoma; MTS#1: retro-splenic metastasis; MTS#2: subhepatic metastases; MTS#3: pelvic metastasis; UC: urothelial carcinoma; UC#1: lymphocyte-enriched component of the urothelial carcinoma; UC#2: lymphocyte-depleted component of the urothelial carcinoma. AD colon adenoma, VAF variant allelic frequency.

Somatic mutational signature prediction from WES data identified the predominance of Signature 15 in all the samples, followed by Signatures 6, 1, and 14 (Fig. 2a and Supplementary Fig. 2a–d). TSO500 targeted panel confirmed these signature profiles (Supplementary Fig. 3).

WES revealed a high number of somatic variants for all the evaluated specimens (Supplementary Table 1), in line with the TSO500 targeted DNA sequencing, in which all of the lesions showed remarkably high TMBs (range: 226–530 mut/Mb, Supplementary Table 1) and high MSI levels (range: 7–52% of unstable loci over the total MS loci available for each sample, Supplementary Table 1). The lowest MSI levels were detected in two metastatic deposits. Interestingly, this ultra-mutator phenotype was also present in the precancerous adenoma (Supplementary Table 1).

We then analyzed private and shared non-synonymous mutations across the different lesions. Both WES and TSO500 Jaccard coefficient analysis showed a tight correlation between the mucinous CRC and the metastatic lesions (Fig. 2b).

This was confirmed also by the unsupervised clustering based on the allele frequency of identified variants in cancer-associated genes by the TSO500 panel, where the primary mucinous colorectal carcinoma and all of the metastatic lesions clustered together, whereas the non-mucinous primary colorectal cancer and the urothelial carcinoma clustered separately (Fig. 2c). Metastatic lesions mostly shared the same variants, with the right pelvic lesion (MTS#3) featuring a higher number of private mutations (Fig. 2c).

A somatic mutation in the *PMS2* gene (i.e., “second hit”) was identified in all of the lesions, including the adenomatous polyp (Supplementary Table 2). The mucinous CRC and the metastatic deposits harbored the same alteration, whereas independent nonsense mutations were found in the other lesions.

Interestingly, targeted sequencing identified four mutations shared by all of the tissue samples, including the urothelial carcinoma: *BRCA1* p.Val340GlyfsTer6, *DICER1* p.Arg490His, *IRS2* p.Arg744Cys, and *PDGFRB* p.Arg853Trp (Supplementary Table 3). The *BRCA1* variant can be classified as pathogenic since the frameshift introduces a premature stop codon, the *DICER1* mutation was predicted potentially damaging by 6/13 algorithms, the *IRS2* p.Arg744Cys by 8/13, and the *PDGFRB* p.Arg853Trp by 11/13 predictors. No contamination between samples was detected by the contamination score calculated by the Illumina pipeline. The seven DNA samples were divided into three independent experiments following DNA re-extraction. Moreover, the four variants were further validated by Sanger sequencing.

Hence, we investigated the possibility of genetic mosaicism in this patient. DNA was extracted from multiple normal tissue samples (multiple buccal swabs, urothelial cells from urine samples, normal colonic mucosa adjacent to CRC, normal colonic mucosa distant from the CRC, normal gastric mucosa) and subjected to ultradeep sequencing with the OCAv3 panel (mean read-depth across samples 15000X) allowing the potential detection of the *PDGFRB* and the *BRCA1* shared variants. However, none of the tested samples harbored any somatic mutations including the *PDGFRB* and *BRCA1* variants.

In the endeavor to provide alternative explanations for the occurrence of these four variants across independent lesions, we followed two approaches. First, we checked the flanking context around the four mutations: only the *BRCA1* mutation falls within a repeated region of “As” (item chr17.176101 from WindowMasker), whereas the other mutations are not located within recognized repeated genomic sequences. Second, we intersected the WES results of the different samples to obtain a list of common variants i.e., mutations shared between any pairs of tumor samples). We observed that genetically dependent tumors (i.e., CRC#1 and the metastatic deposits) shared ~7000 somatic variants. Conversely, completely independent lesions (i.e., CRC#1, CRC#2, and UC) shared a 75-fold times smaller, yet non negligible, number of somatic variants across any tumor pairs (range: 88–112) (Supplementary Table 4). Of note, even the preinvasive lesion of the colorectal adenoma and the urothelial carcinoma shared 51 variants. This scenario is quite unique given the distinct nature of the tumoral lesions, particularly when considering the different origins of the tumors (colon versus bladder) and the type of lesion (primary tumors versus metastases). As expected, we found a decrease in the number of shared variants when considering multiple lesions, up to the four mutations originally found as common to all lesions (Supplementary Table 5). Interestingly, the VAF of the mutations shared across lesions significantly decreased at the increase of the number of lesions (Supplementary Fig. 4). This indicates the subclonal nature of variants that were common to a high number of lesions. Together these data suggest that these four variants may be a consequence of the hypermutator phenotype of these tumors.

### Clinical significance of somatic mutations

Variants from the TSO500 sequencing were classified by using OncoKB^15^. The shared *BRCA1* p.Val340Glyfs*6 variant showed a 3B level. CRC#1 showed the *PIK3CA* p.Arg88Gln with a 3B level. A frameshift *ATM* variant (p.Lys2811SerfsTer6) with a 3B level was shared by the mucinous CRC and corresponding metastatic deposits. No variants related to resistance to currently approved drugs were described for primary CRC and metastatic lesions. The urothelial carcinomas harbored three private mutations with specific annotation, two *KRAS* mutations (p.Gly13Asp and p.Ala146Thr) with resistance level R1, and the level 4 *NF1* p.Arg2637Ter. Mutations affecting *PIK3CA* residue 88 and *KRAS* residue 146 have been described as overrepresented in the context of a *POLE* mutagenic pressure^16^.

### Gene expression analysis

Samples preferentially clustered according to the tissue of origin when gene expression targets were grouped in pathway scores (Fig. 2d). We then assessed whether the lesions displayed a different content of immunological markers. All of the metastatic deposits expressed higher levels of markers for total TILs compared to primary CRCs. When comparing the relative amount of each cell subtype with the total TILs, we observed that the urothelial carcinoma showed a higher level of CD8 exhausted cells than the other lesions; in addition, among the CRC metastatic samples MTS#3 displayed a T cell exhaustion level comparable to that found in the urothelial tumor (Fig. 2e). Urothelial cancer presented a higher level of mast cells, NK cells, and Treg (Fig. 2e), whereas the CRC lesions displayed a general intermediate and homogeneous level for all immune cell markers.

When comparing the differentially active pathways across lesions, the urothelial tumor had lower levels for antigen presentation, activation of cytokines and chemokines, interferon signaling, and JAK-STAT pathway activation compared to the others. We also detected a high level of genes associated with proliferation. The proliferation score was very low in the two metastatic lesions responsive to immunotherapy, whereas reached a significantly higher value in the lesion less responsive to treatment (MTS#3, Fig. 2e).

## Discussion

This report focused on the germline and somatic genetic characterization of a young patient affected by a bifocal colorectal carcinoma with peritoneal progression, treated with nivolumab + anti-LAG3 with good results. The study aimed to define the potential influence of variants at the germinal level, to explain both the juvenile onset of cancer and the good response to therapy. The transcriptomic landscape of the different lesions was characterized in the attempt to complement the interpretation of possible determinants behind the differential response to immunotherapy of distinct lesions and the development of a second tumor, of urothelial origin, during immunotherapy treatment.

WES identified a *PMS2* splicing variant with a known pathogenic effect and a germline variant of the *POLE* gene with a likely pathogenic effect (p.Ser297Cys *POLE*) affecting a region encoding for the exonuclease domain in which both germinal and somatic pathogenic mutations have been previously described^5,17^. The paternally inherited *PMS2* c.2174 + 1 G > A is extremely rare in the global population, not reported in the phase3 of the 1000 genome project^18^ and in NHLBI GO Exome Sequencing Project (ESP) (http://evs.gs.washington.edu/EVS/), with only four heterozygous cases over the 122.207 in the gnomAD database^19^. The variant was confirmed to induce aberrant transcription by Van der Klift et al., describing the production of a minor transcript with the exon 12 skipping and a major one composed by a partial intron sequence in the HEK292 cells (Δ12 + ▾12q_421nt)^11^. The variant is defined pathogenic by the ClinVar database with accession number VCV000091329.19 and is associated with Lynch syndrome^20^.

When dissecting the p.Ser297Cys *POLE* variant, the strong retention of the residue within the species associated with the maintained stability of the protein led to hypothesize that this alteration may be pathogenic. This was corroborated by the prediction of deleterious significance rendered by 11/14 predictor tools and by the transmission from the maternal lineage, affected by colorectal polyps and with a family history of CRC, uterus, and pancreatic cancers. The pathogenic nature of this variant was demonstrated by the revertant assay in *Saccharomyces cerevisiae*, with a strong increase of revertant colonies in the mutated clones. The effect of the variant is similar to other *POLE* mutations described by Castellsagué et al., in which the p.Thr278Lys showed a similar reverting effect, stronger than the p.Leu424Val mutants^21^.

*POLE* missense variants are associated with the polymerase proofreading-associated polyposis (PPAP), a known autosomal dominant hereditary disease associated with the development of polyps and an increased risk of cancer at different sites. *POLE* variants appear to be enriched in juvenile cancers^22^, with the youngest cases being diagnosed with polyps and cancer at 16 and 27 years of age^23^.

The proband genotype can explain the unusual clinical and familiar history. Although none of the relatives developed tumors at a very young age, the proband can be considered an exceptional phenotype stemming from the oncogenetic pressure of a germline coexistence of pathogenic MMR and *POLE* variants, thus explaining the primary tumors developed before the age of twenty. The clinical scenario here described defines a “Multilocus Inherited Neoplasia Alleles Syndrome (MINAS^24^)” that could be labeled as “POLE-LYNCH” collision syndrome.

The features emerging from the somatic genetic landscape analysis reflect the germline scenario. The ultra-mutator phenotype is proper of a *POLE* mutation, in both germline and somatic settings^25^. The *PMS2* mutation per se would not explain such a high number of variants. Indeed, TMB levels around 20/40 mut/Mb are typically reported^26^. Of note, a much higher TMB (>200 mut/Mb) has been documented in a germline *PMS2* variant carrier with a somatically acquired *POLE* mutation^27^.

Furthermore, in all of the lesions, a predominance of Signature 15 and Signature 6 followed by Signature 1 (related to “aging”) and Signature 14 was demonstrated. Signature 15, similarly to Signature 6 (related to defective DNA MMR), contributes a very large number of substitutions and small indels at nucleotide repeats, however, it exhibits greater prominence of C > T at GpCpN trinucleotides. Signature 15 has never been reported outside small cell lung cancer and gastric cancers^28^. Previous reports^21,29,30^ have showed that Signature 14 characterizes tumors with mutations in *POLE* and inactivation of MMR. One could have expected this signature as most prevalent in this context as well, however, it should be noted that these studies did not report a concomitant, inherited alteration, rather the occurrence of a germline MMR deficiency and a somatic *POLE* alteration.

As part of the molecular analysis, we identified four somatic variants shared by all the precancerous and cancerous lesions, including a frameshift affecting *BRCA1*. Sample contamination was excluded. On one hand, this finding may suggest an event occurred early during development, however, multiple samples pertaining to normal tissue components were wild-type for these variants at ultradeep sequencing. Nevertheless, we acknowledge that we cannot completely rule out the presence of mosaicism due to the difficulty of detection stemming from the intrinsic nature of the mosaic per se^31^. On the other hand, the relatively high number of variants that were detected in common across multiple samples, with an exponential increase when considering only two samples (e.g., one of the CRCs and the urothelial carcinoma, two distinct neoplastic lesions of different origin), more likely prompts to consider that the exceptionally high hypermutability originated by this POLE-LYNCH collision syndrome may have led to the co-occurrence of these variants on a stochastic basis.

Finally, the transcriptomic analyses may shed light on some open questions related to ICI treatment. This patient showed a good, yet not exquisite, sensitivity to the nivolumab plus anti-LAG3 combination. The differential response of one of the metastases and the occurrence of a second primary tumor over treatment may prompt important questions. Gene expression analysis showed relatively reduced activation of the immune system in the urothelial carcinoma, which was also associated with reduced activation of the cancer-dependent immune-activating pathways compared to the other tumoral lesions, despite the presence of a very high TMB. This may be associated with the early diagnosis and surgery of the urothelial lesion; however, one could also hypothesize that the low immune cell content may have facilitated a kind of immune escape.

The three metastatic lesions showed an immunotherapy-influenced gene expression profile, featuring the highest level of both total and reactive TILs, and low levels of expression of cell proliferation-associated genes. The metastasis less sensitive to immunotherapy (MTS#3) was enriched in exhausted CD8. The accumulation of these hyporesponsive T-cells during ICI treatment has been associated with poor responses and therapy resistance^32,33^.

In conclusion, this thorough molecular characterization of a single patient with multiple tumor lesions unveiled the co-occurrence of germline *POLE* and *PMS2* pathogenic variants (from the maternal and paternal lineage, respectively) predisposing to the development of multiple neoplasms.

The p.Ser297Cys *POLE* variant is a mutation never described before and documentation of its pathogenicity has been functionally provided in this study.

The ultra-mutator genotype and MSI status generated by the contribution of these germline alterations contextualize the response to immunotherapy experienced by the patient. Although we can only speculate on the etiopathogenesis of the second primary tumor of urothelial origin, we document a kind of “native” immune refractoriness of this lesion.

The inherited predisposition was identified by comprehensive genomic profiling, pursued based on the unique clinical history of the patient and on the open questions following the initial genetic counseling run on a limited number of CRC predisposing genes.

## Methods

The Patient signed a specific informed consent (PROFILING #001-IRCC-00IIs-10, protocol approved by the Ethical Committee of FPO-IRCCS Candiolo Cancer Institute), which allowed to proceed with an in-depth genomic investigation of tumor and normal tissue samples. Relatives of the proband signed informed consent for diagnostic germline molecular analyses performed at the Medical Genetics Unit of AOU Città della Salute e della Scienza in Turin. The proband and the relatives were notified that the results of this work would have been published.

### Tissue sample review, DNA, and RNA extraction

Formalin-fixed paraffin-embedded (FFPE) tissue samples corresponding to two synchronous primary CRCs (CRC#1: mucinous adenocarcinoma, CRC#2: non-mucinous adenocarcinoma), a single adenomatous polyp (AD), three metastatic deposits of intestinal origin (MTS), and urothelial carcinoma (UC) were retrieved (Fig. 1a). The patient presented four metastatic lesions that were surgically resected, however, one of the lesions could not be analyzed since the FFPE block was externalized for Central Laboratory review, hence the remaining three lesions were profiled. Corresponding Hematoxylin and eosin (H&E) sections were reviewed to assess tumor cellularity and eight-micron thick sections were cut, stained with H&E, and microdissected under a stereomicroscope to enrich for tumor cell content, as previously described^34^. DNA was extracted using the GeneRead DNA FFPE Kit (Qiagen, Hilden, Germany), RNA was extracted with the High Pure FFPET RNA Isolation Kit (Roche, Basel, Switzerland). For gene expression analysis, two components of the urothelial carcinoma were separated by microdissection, one more enriched in lymphocytic infiltration (UC#1) than the other (UC#2).

To assess potential mosaicism, we purified DNA as described above from FFPE normal colonic mucosa (both CRC adjacent and distant), from normal gastric mucosa, and from cell blocks of urine samples. Blood draws and buccal swabs were collected from the patient and DNA was purified using the QIAamp DNA Mini Kit (Qiagen, Hilden, Germany).

DNA and RNA samples were quantified with both spectrophotometric (Nanodrop 1000, Thermo Fisher Scientific, Waltham, MA, US) and fluorometric (Qubit, Thermo Fisher Scientific, Waltham, MA, US) assays. The integrity of the RNA was also assessed by the BioAnalyser 6000 Nano Assay on the BioAnalyzer 2100 instrument (Agilent, Santa Clara, CE, US).

### Immunohistochemistry

Three-micron thick sections were cut from FFPE blocks of the primary CRCs and subjected to immunohistochemistry for MMR proteins using the monoclonal antibodies anti-MLH1 (clone G168-15, 1:40 diluted, BD Biosciences, Heidelberg, Germany), anti-MSH2 (clone FE11, 1:50 diluted, Calbiochem, Merck, Darmstadt, Germany), anti-MSH6 (clone 44, 1:200 diluted; BD Biosciences), and anti-PMS2 (clone A16-4, 1:40 diluted, BD Biosciences).

Immunohistochemistry was performed on a Leica Bond Autostainer (Leica microsystems).

### Next-generation sequencing analysis

The whole exome was captured from genomic DNA using the Illumina Exome Panel (CEX) (45 Mb, Illumina, San Diego, California, USA) following the Illumina DNA Prep with Enrichment protocol (Illumina, San Diego, California, USA). Briefly, 800 ng of genomic DNA was fragmented and then linearly amplified and indexed with nine cycles of PCR. PCR products were hybridized with the bait library and the captured library was amplified with ten PCR cycles. Libraries were sequenced using Illumina NovaSeq6000 (Illumina, San Diego, California, USA) in 150nt-long paired-end modality.

Germline and somatic mutations were identified with a previously published pipeline^35^, as implemented in the HaTSPiL framework^36^. In particular, sequencing reads from each sample were aligned to the human genome reference (GRCh37/hg19) using Novoalign (http://www.novocraft.com/) with default parameters. At most three mismatches per read were allowed and PCR duplicates were removed using the Picard Markduplicates tool (https://broadinstitute.github.io/picard/). Local realignment around indels was performed using GATK RealignerTargetCreator and IndelRealigner tools (https://gatk.broadinstitute.org/hc/en-us). Single nucleotide variants (SNVs) and small insertion/deletions (InDels) were identified using MuTect v.1.1.17 (https://software.broadinstitute.org/cancer/cga/mutect), Strelka v.1.0.14^37^, and Varscan2 v.2.3.6 (http://varscan.sourceforge.net/). Only variants identified as “KEEP” and “PASS” in MuTect and Strelka, respectively, were considered. SNVs and InDels were retained if (i) had allele frequency ⩾5%, (ii) in a genomic position covered by at least ten reads, and (iii) had at least 1% of supporting reads mapping on both DNA strands. In the tumor sample, SNVs and InDels were identified as somatic if absent in the normal counterpart. Furthermore, only somatic SNVs that were identified by two out of three variant callers were considered. ANNOVAR was used to identify non-silent (non-synonymous, stopgain, stoploss, frameshift, nonframeshift, and splicing modifications) mutations using RefSeq v.64 as a reference protein dataset. SNVs and InDels falling within 2 bp from the splice sites of a gene in one of the three datasets were considered as splicing mutations.

The VCF files obtained from the whole blood sequencing were then scanned for pathogenicity using the InterVar tool, a command-line bioinformatics software for clinical interpretation of genetic variants according to the ACMG2015 guidelines^38^.

The following public databases were used for variant interpretation: ClinVar (https://www.ncbi.nlm.nih.gov/clinvar/), Ensembl (https://www.ensembl.org/index.html), Varsome (https://varsome.com/), InSIGHT (https://www.insight-group.org/variants/databases/), LOVD (https://databases.lovd.nl/shared/genes), and the Human Genome Mutations Database (HGMD, http://www.hgmd.cf.ac.uk/ac/index.php). All variants were filtered using a minor allele frequency (MAF) cut-off of less than 0.01% in the gnomAD and dbSNP databases. The pathogenicity of all variants was then assessed by 13 in silico prediction tools using ANNOVAR tools^39^. Clinical significance was confirmed with the Alamut software. The effect on the 3D structure of the protein of variants was evaluated using the Phyre2 software and the related SusPect pathogenicity algorithm. All variants are described in accordance with the recommendations of the Human Genome Variation Society (http://www.hgvs.org/mutnomen).

The similarity of somatic mutation spectra across tumors was calculated using the Jaccard Coefficient (JC) as previously described^35^. In particular, for each tumor, JC was measured as the proportion of shared somatic mutations over the total number of somatic mutations.

To characterize a specific pattern of nucleotide substitutions, present in each tumor, mutational signatures were calculated using the *estimateSignatures* and *extractSignatures* functions with default parameters from the Maftools R packages (https://github.com/PoisonAlien/maftools). The extracted signatures were compared to known signatures, and proposed etiology, from COSMIC database v.2 (https://cancer.sanger.ac.uk/signatures/signatures_v2/).

DNA sequencing was applied to a total of 40 ng using the Illumina TruSight Oncology 500 panel (panel size: 1.94 Mb, encoding 1.2 Mb; Illumina, San Diego, California, USA), which includes 523 cancer-related genes. MSI status of ~120 loci and TMB can be assessed. Following the manufacturer’s protocol, the generated libraries were sequenced on the Illumina Next-Seq 500 instrument (Illumina, San Diego, California, U.S). The Illumina local app associated with the TSO500 panel was used to perform sequencing and contamination QCs and to generate fastq files through alignment to the human reference sequence GRCh37 (hg19). The Illumina App performed variant calling without a normal tumor pipeline, based on querying different genetic databases and taking into account variant allele frequency (VAF). However, we confirmed the germline-only variants by comparing the results of non-tumorigenic and neoplastic DNA sources.

We annotated the somatic variants using the previously defined ANNOVAR tools for pathogenicity. To identify clinical significance, the OncoKB^15^ annotation was performed using the command line python tool oncokb/oncokb-annotator. As for the TMB, we reported three different values: (i) the TSO500 TMB, calculated as the number of all, non-synonymous, somatic variants/Mb provided by the pipeline, (ii) a custom TMB which comprises all the non-synonymous, somatic variants with a VAF >5% (nonsTMB), and (iii) a custom TMB which comprises all the somatic variants, both synonymous and non-synonymous, with a VAF >5% (sTMB). The TMB values were calculated over the real, sequenced region for each sample.

The OCAv3 target gene panel (161 genes, Thermo Fisher Scientific, Inc.) was applied to germline DNA from the blood of both the proband and the parents. Moreover, the same panel was applied for the mosaic ultradeep sequencing.

Briefly, a total of 40 ng of DNAs was used to build libraries using the Ion AmpliSeq™ Library kit Plus (Thermo Fisher Scientific, Inc.) according to the manufacturer’s instructions. Ion Xpress Barcode and Ion P1 Adapter (Thermo Fisher Scientific, Inc.) were added during the library preparation. The final amplicon libraries were quantified using the Ion Library TaqMan™ Quantitation Kit (Thermo Fisher Scientific, Inc.) and diluted to 50 pM for the subsequent sequencing. Template generation and chip loading were performed using The Ion Chef™ System (Thermo Fisher Scientific, Inc.) using the Ion 540™ Kit – Chef. The obtained libraries were finally sequenced using the Ion GeneStudio^TM^ S5 Plus System using the ion 540 Chip kit (Thermo Fisher Scientific, Inc.). BAM files derived from processed raw data were generated by the Ion Torrent platform-specific pipeline software. All the BAM files were transferred on the Ion Reporter Software (v. 5.10.5.0) (Thermo Fisher Scientific, Inc.) and analyzed by the Oncomine OCAv3 w3.0 - DNA - Single Sample (v. 5.10). The Ion Reporter workflow was applied to identify SNVs, indels, and CNVs using the preset parameters.

### MSI analysis via PCR based tests

All purified DNAs were also analyzed for MSI status by using the MSI analysis system—version 1.2 (Promega, Madison, WI, USA), which includes five mononuclear and monomorphic microsatellite loci, and by using the Titano MSI panel (Diatech Pharmacogenetics, Jesi, Italy), which includes five mononuclear (BAT25, BAT26, BAT40, NR21, and NR24) and five dinuclear (D2S123, D17S250, D5S346, D18S58, and TGFβRII) target markers, following the manufacturer’s protocol.

Products were analyzed by capillary electrophoresis using an ABI 3100 Genetic Analyzer (Applied Biosystems, Foster City, CA, USA). Samples were considered MSI when harboring >40% unstable loci^40^.

### MLH1 methylation analysis

The somatic methylation status of the *MLH1* promoter was assessed by using combined bisulfite conversion-pyrosequencing as previously described^41^. Briefly, 200 ng of DNA were converted using the MethylEdge Bisulfite Conversion System (Promega, Madison, WI, USA) following the standard protocol. Fifty ng of converted DNA were then amplified and sequenced using the protocol described here^41^. The mean percentage of the C in 5 CpG dimers was used to assess the *MLH1* methylation status of the samples.

### Sanger sequencing

To validate the four shared variants, we designed 4 PCR assays to amplify the region encompassing these mutations. Primers for amplification, protocol, and thermal profile are reported in Supplementary Table 6. The PCR products were subjected to sequencing PCR reaction using the BigDye Terminator v3.1 Sequencing kit (Applied Biosystems, Waltham, Massachusetts, USA), using the following parameters: denaturation at 98 °C for 2 min, followed by 25 cycles of 96 °C for 10 s, annealing at 50 °C for 5 s and extension at 60 °C for 4 min. Sanger sequencing products were separated with the capillary electrophoresis in a 3730 Genetic Analyzer (Applied Biosystems, Waltham, Massachusetts, USA).

### Gene expression analysis

Targeted gene expression was carried out by using the PanCancer IO 360™ Panel (NanoString Technologies, Seattle, WA, USA) on the cancer lesions. At least 100 ng of total RNA were incubated for 14 h at 56 °C with probes encompassing a total of 770 genes. Captured targets were then purified and immobilized with the NanoString nPrep Station and scanned with the NanoString nCounter instrument (NanoString Technologies, Seattle, WA, USA). The RCC count files were normalized and analyzed with the nSolver Software (NanoString Technologies, Seattle, WA, USA) and characterized with the nSolver Advanced Analyses, based on robust R statistics. We considered in particular the pathway score (calculated as the first principal component of the pathway genes normalized expression) and the cell type score, as previously reported^42^.

### Yeast assay

Wild-type *POL2* and *pol2*-S312C mutant alleles were cloned under the control of their own promoter into YCplac33. A 2.5 Kb SacI/KpnI fragment—carrying the promoter of *POL2* and the *POL2* coding sequence up to the KpnI site—and a 5.5 Kb KpnI/SacI fragment containing the *POL2* coding sequence from the KpnI site to the STOP codon—were PCR amplified with the Pol2FW/Kpn_RV and Kpn_FW /Pol2RV oligo pairs respectively. The PCR fragments, cut with KpnI/SacI, were cloned into YCplac33 KpnI/SacI digested. The p.Ser312Cys point mutation was introduced in the plasmid carrying the 2.5 Kb fragment using the Quick-Change Xl Site-Directed Mutagenesis Kit (Agilent Technologies) with the Cys_FW and Cys_RV primers. To obtain the full-length wild-type and mutant variant of Pol2 the 2.5 Kb wt and *pol2-*S312C fragments were cloned into the construct carrying the 5.5 kb fragment. All constructs were verified by sequencing. The ability of the cloned variants to rescue *pol2Δ* lethality was assessed by a complementation assay testing viability in haploid strains.

By homologous recombination, using a PCR fragment amplified with the Pol2D_F and Pol2_R oligos^43^, we obtained the *POL2*/*pol2*-S312C heterozygous diploid (Ry10863). The diploid was next transformed with yeast centromeric plasmids either empty or carrying wild-type and mutant *POL2*, p*CEN-URA3*, p*CEN-POL2-URA3*, and p*CEN-pol2-S312C-URA3* plasmid, respectively. The diploids were sporulated and microdissected to^44^ obtain the haploids carrying the genotype of interest. Both haploid and diploid strains were assayed for their ability to promote revertants. About 2 × 10^7^ cells were plated into tryptophan lacking media. Plates were scored after 5 and 10 days. All strains used in this study are isogenic to W303 and listed in Supplementary Table 7A. Oligos, primers, and plasmids are available in Supplementary Table 7B and 7C.

### Reporting summary

Further information on research design is available in the Nature Research Reporting Summary linked to this article.

## Supplementary information

Supplementary Material

REPORTING SUMMARY

## Data Availability

Sequencing data are not publicly available in order to protect patient privacy. The ethics committee and the informed consent does not allow for these data to be deposited into a secure access controlled repository. Qualified researchers can apply for access to the data by contacting the corresponding authors. The data will be made available on reasonable request.
