# Supplementary material for: Collision of germline POLE and PMS2 variants in a young patient treated with immune checkpoint inhibitors
Source: NPJ Precis Oncol. 2022 Mar 8;6:15. doi: 10.1038/s41698-022-00258-8 (PMC8904527; doi:10.1038/s41698-022-00258-8)
Supplement: Supplementary file 1 — Supplementary Material [file 41698_2022_258_MOESM1_ESM.pdf]

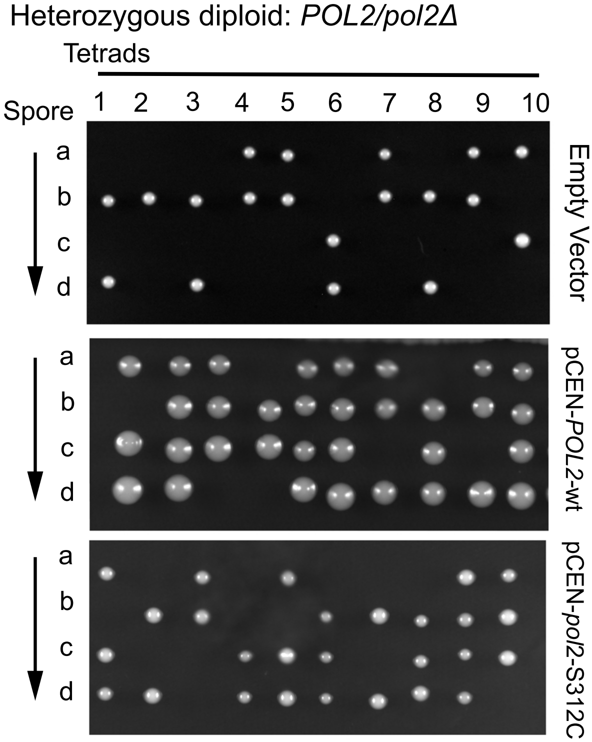

Supplementary Figure 1. Complementation assay. Tetrad analysis of an heterozygous *POL2/pol2Δ* diploid carrying an empty vector or centromeric plasmids with WT or pol2-S312 allelic variant

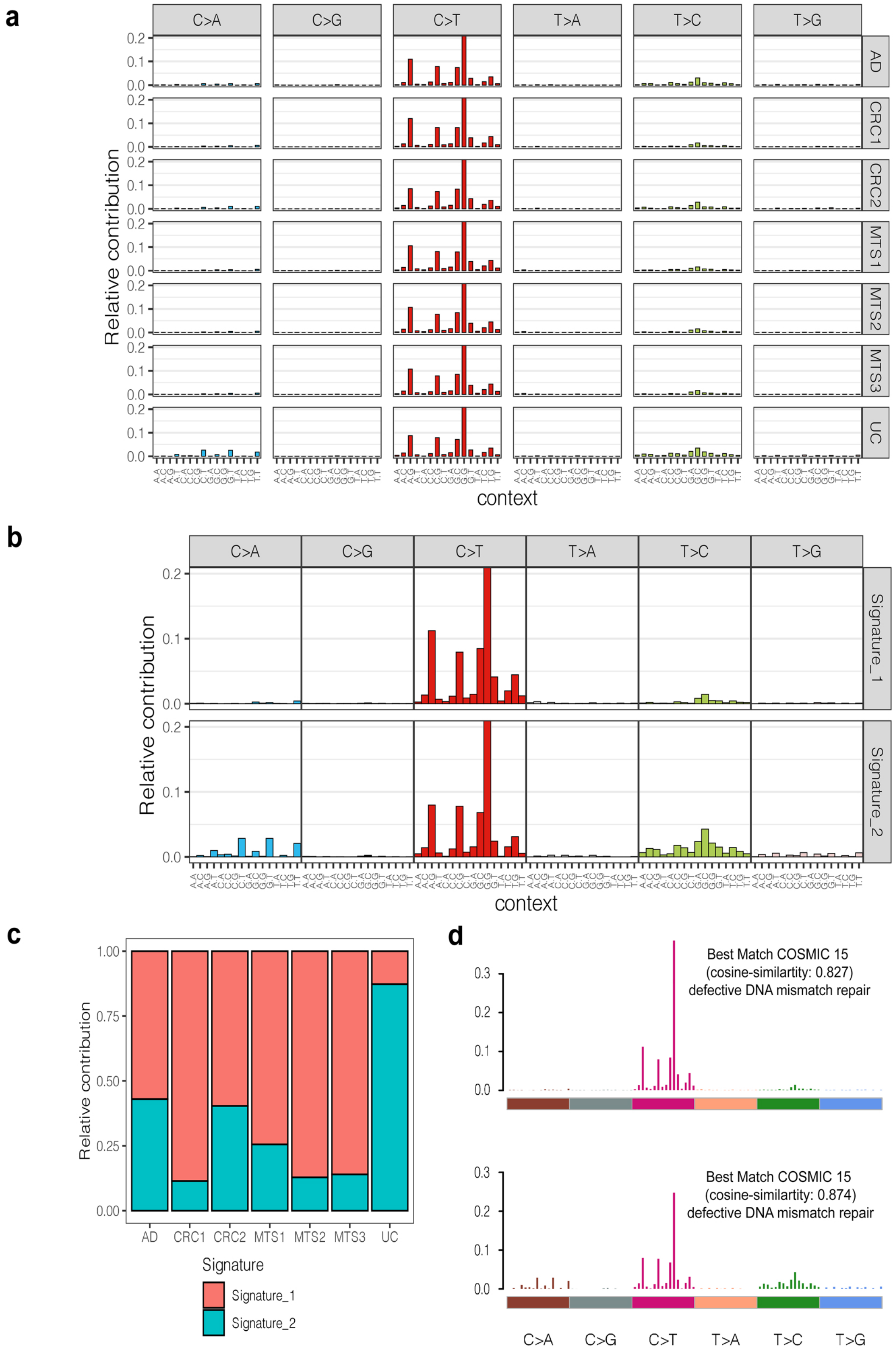

Supplementary Figure 2. Deciphering Signatures of Mutational Processes. a. Quantification of nucleotide substitutions in the trinucleotide contexts for all samples. Six main substitution categories on the x-axis represent the different mutations (C>A, C>G,... ,T>G). Each category is splitted additional 16 categories to represent the combinations of bases that can prefix and postfix the mutation.**b.**The two most representative signatures of mutational processes. **c.** Barplot represents the contribution of two most representative sign**a**tures of mutational processes in each sample. **d.** The most similar COSMICv2 signatures obtained using the plotSignatures function from the maptools R package**.**

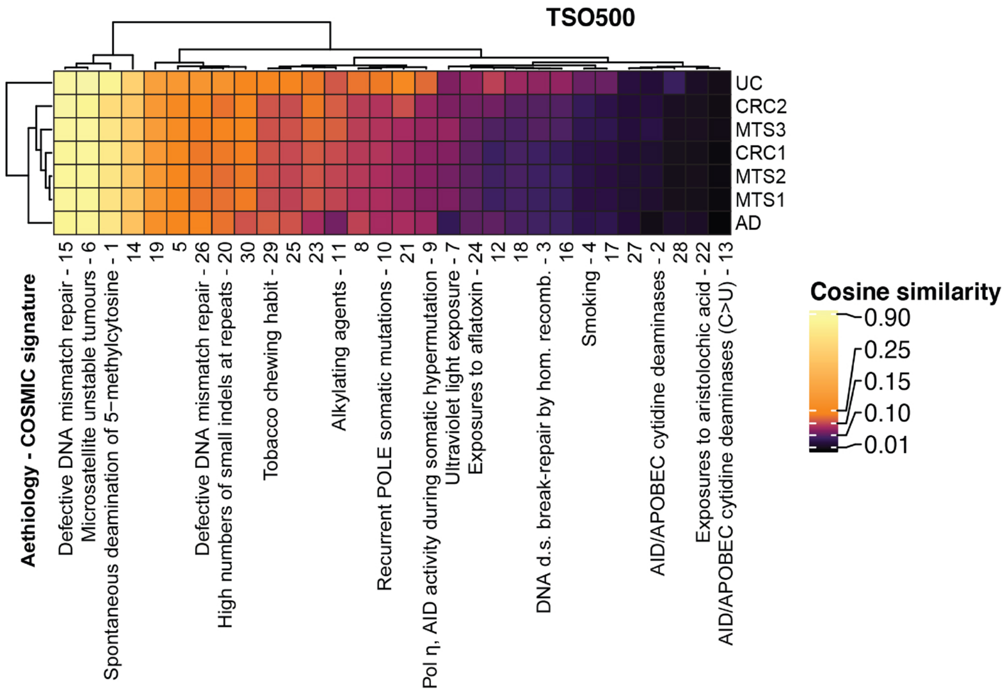

Supplementary Figure 3.Mutational signature results exploiting data from TSO500 targeted sequencing. The heatmap chart shows the prevailing signatures 15, 6, 1, and 14 in all the sequenced lesions. The results are in line with the signatures defined by WES.

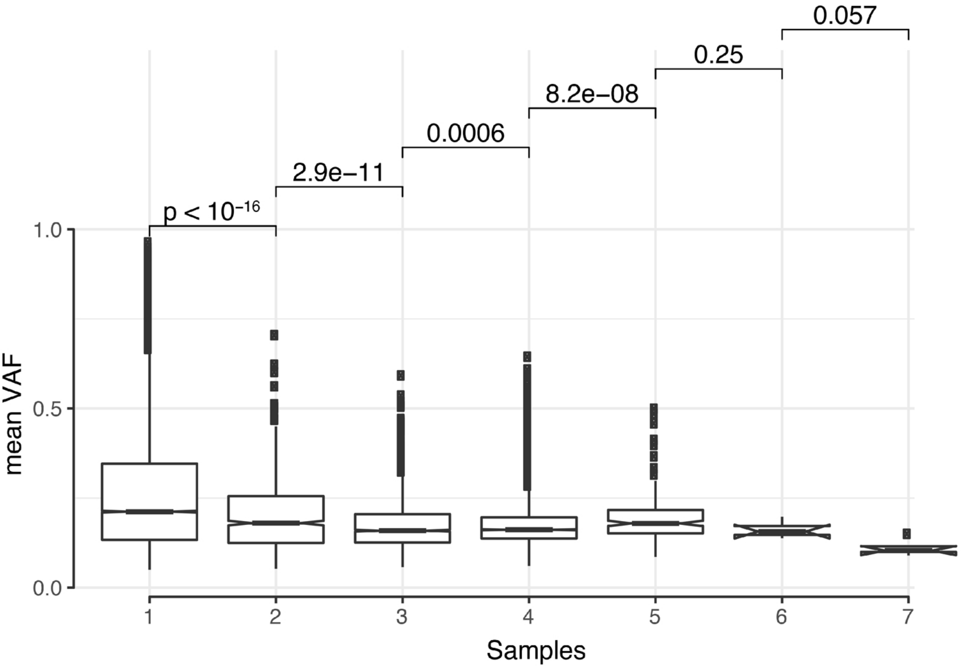

Supplementary Figure 4. VAF distribution plots (percentile distrubution). The allele frequency of the shared variants decreases at the increaseof the intesected samples, suggesting subclonality of the shared variants

Supplementary Table 1. TMB values and MSI status for all of the samples.

	# WES variants	TMB TSO500	sTMB	nonsTMB	# Ustable Loci	% Unstable Loci
CRC#1	9154	530	760	385	58	52
CRC#2	6221	295	476	226	31	28
MTS#1	9588	414	541	296	13	12
MTS#2	8973	467	620	330	7	7
MTS#3	9351	305	483	260	45	50
UC	9703	413	572	320	31	27
AD	5101	226	324	201	51	41

CRC # 1: mucinous colorectal cancer; CRC # 2: colorectal cancer without mucinous features; MSI: microsatellite instability; MTS# 1: retro-splenic metastasis; MTS# 2: subhepatic metastases; MTS# 3: pelvic metastasis; UC: urothelial carcinoma, AD: adenoma; TMB: tumor mutation burden; WES: whole exome sequencing.

Supplementary Table 2. Somatic second hit affecting *PMS2*.

	Gene	Germline Mutation	VAF	Somatic Mutation	VAF
CRC#1	*PMS2*	c.2174+1G>A	0.38	p.Lys447Ter	0.31
CRC#2	*PMS2*	c.2174+1G>A	0.42	p.Asp414ArgfsTer42	0.10
MTS#1	*PMS2*	c.2174+1G>A	0.34	p.Lys447Ter	0.29
MTS#2	*PMS2*	c.2174+1G>A	0.31	p.Lys447Ter	0.15
MTS#3	*PMS2*	c.2174+1G>A	0.34	p.Lys447Ter	0.14
UC	*PMS2*	c.2174+1G>A	0.39	p.Gln77AlafsTer4	0.40

CRC # 1: mucinous colorectal cancer; CRC # 2: colorectal cancer without mucinous features; MTS # 1: retro-splenic metastasis; MTS # 2: subhepatic metastases; MTS # 3: pelvic metastasis; UC: urothelial carcinoma, VAF: Variant Allele Frequency

Supplementary Table 3. Features of the four variants shared by all the lesions.

Gene	Coding	Protein	CRC # 1	CRC # 2	MTS # 1	MTS #2	MTS #3	UC	AD	Pathogenic Prediction	Onco KB level
BRCA1	c.1016dupA	p.Val340GlyfsTer6	41%	26%	29%	16%	34%	40%	24%	*	none
DICER1	c.1469G>A	p.Arg490His	35%	27%	18%	16%	31%	40%	26%	46%	none
IRS2	c.2230C>T	p.Arg744Cys	34%	29%	18%	12%	22%	36%	30%	62%	none
PDGFRB	c.2557C>T	p.Arg853Trp	30%	27%	17%	11%	25%	35%	29%	84%	none

* stop loss variant is considered as damaging
Pathogenic prediction is calculated as the number of pathogenic values over the total tested algorithm
CRC # 1: mucinous colorectal cancer; CRC # 2: colorectal cancer without mucinous features; MTS # 1: retro-splenic metastasis; MTS # 2: subhepatic metastases; MTS # 3: pelvic metastasis; UC: urothelial carcinoma, AD: adenoma.

Supplementary Table 4. Number of shared variants by intersecting pairs of samples.

Sample 1 ID	Sample 2 ID	# of Shared Variants
CRC#1	CRC#2	112
CRC#1	AD	105
CRC#1	UC	88
CRC#1	MTS#3	7902
CRC#1	MTS#1	7481
CRC#1	MTS#2	7634
CRC#2	AD	68
CRC#2	UC	88
CRC#2	MTS#3	113
CRC#2	MTS#1	105
CRC#2	MTS#2	104
AD	UC	51
AD	MTS#3	96
AD	MTS#1	95
AD	MTS#2	94
UC	MTS#3	89
UC	MTS#1	85
UC	MTS#2	84
MTS#3	MTS#1	8367
MTS#3	MTS#2	8626
MTS#1	MTS#2	8131

Supplementary Table 5. Number of shared variants depending on the number of intersected lesions.

Combination of Samples	# of Mutations
1	34146
2	10301
3	9160
4	6667
5	208
6	8
7	4

Supplementary Table 6. PCR protocol and primer sequence for Sanger validation of the four variants shared by all of the lesions.

Variant	Primer	Primer Sequence	Thermal Profile
IRS2 p.Arg744Cys	F	5'-CACGTACTGGTCGCTG-3'	95°C x 3 min<br>**30 cycles of**<br>95°C x 30 sec<br>58° x 30 sec<br>72° x 45 sec<br>72°C x 5 min
	R	5'-AGGACAGTGGGTACATGC-3'	
DICER1 p.Arg490His	F	5'-GGAGACCCTATGGGCACTTTG-3'	
	R	5'-GTTTATTTTCTTTCAGCAACGTTTAACTTCC-3'	
BRCA1 p.Val340GlyfsTer6	F	5'-TTAGCAAGGAGCCAACATAAC-3'	
	R	5'-TCCATTCTTTTCTCTCACACAG-3'	
PDGFRB p.Arg853Trp	F	5'-ATCCTGTCCCTGCACACATAG-3'	
	R	5'-AAGCTGGTCAAGATCTGTGACTTT-3'	

Supplementary Table 7a. Clone strategy of the study.

**Strain**	**Relevant Genotype**	**Reference**
Ry10862	*MATa/MATalpha, ade2-1, leu2-3, ura3, trp1-1, his3-11,15, can1-100, GAL, PSI+*	This study
Ry10863	*MATa/MATalpha, pol2::hphM6X/POL2*	This study
Ry10864	*MATa/MATalpha, pol2::hphM6X/POL2, pCEN-URA3 (*Ycplac33)	This study
Ry10865	*MATa/MATalpha, pol2::hphM6X/POL2, pCEN-POL2-URA3*	This study
Ry10866	*MATa/MATalpha, pol2::hphM6X/POL2, pCEN-pol2*-S312C-*URA3*	This study
Ry10868	*MATa, POL2, , pCEN-URA3 (*Ycplac33) Clone1	This study
Ry10869	*MATa, POL2, , pCEN-URA3 (*Ycplac33) Clone2	This study
Ry10870	*MATa, pol2::hphM6X, , pCEN-POL2-URA3* Clone1	This study
Ry10871	*MATa, pol2::hphM6X, , pCEN-POL2-URA3* Clone2	This study
Ry10872	*MATa, pol2::hphM6X, , pCEN-pol2*-S312C-*URA3* Clone1	This study
Ry10873	*MATa, pol2::hphM6X, , pCEN-pol2*-S312C-*URA3* Clone2	This study

Supplementary Table 7a. Oligonucleotides used in the study

Oligo	Sequence 5' – 3'	Note
Pol2D_F	AGCACATTCTATCAAGATAACACTCTCAGGGGACAA GTAT***CGGATCCCCGGGTTAATTAA***	Use with Pol2D_R to delete *POL2.* In bold/italics the sequence annealing in the plasmid
Pol2D_R	GGTAAAGAGGCCATTGAACCTCGCGTTATATACTGC TTAC***GAATTCGAGCTCGTTTAAAC***	Use with Pol2D_F to delete *POL2.* In bold/italics the sequence annealing in the plasmid
Pol2FW	CGCG***GAGCTC***GATCAAAAGATACAAGCCAAGC	FORWARD Anneals at -998bp. Use to amplify the *POL2* promoter. In bold/italics the SacI site
Pol2RV	GCGC***GAGCTC***GAAAATACTCTTTTTCCTTACC	REVERSE Anneals after the STOP codon. In bold/italics the SacI site
Kpn_FW	GAAAG***GGTACC***GGTACTTTGTGTGAAATG	FORWARD Anneals at KpnI site internal at *POL2*. In bold/italics the KpnI site
Kpn_RV	CATTTCACACAAAGTACC***GGTACC***CTTTC	REVERSE Anneals at KpnI site internal at *POL2*. In bold/italics the KpnI site
Cys_FW	CCGCCGTAGATCAAATAATGATGATT***TGC***TATATGAT CGATGGGGAAGG	FORWARD Anneals at +908. In bold/italics the mutated codon
Cys_RV	CCTTCCCCATCGATCATATA***GCA***AATCATCATTATTT GATCTACGGCGG	REVERSE Anneals at +956. In bold/italics the mutated codon

Supplementary Table 7c. Plasmid used in of the study.

Plasmid	Relevant Genotype	Reference
Rp225	YCplac33 (a.k.a. *pCEN-URA3)*	44
Ry714	YCplac33 containing the *POL2* wild type allele under the control of its own promoter (a.k.a. *pCEN-POL2-URA3)*	This study
Ry715	YCplac33 containing the *pol2*-S312C allelic variant under the control of its own promoter *(*a.k.a. *pCEN-pol2*-S312C-*URA3)*	This study
